# The New Medical Practice Act—The Mixed Board Bill

**Published:** 1907-05

**Authors:** 


### THE NEW MEDICAL PRACTICE ACT—THE MIXED BOARD BILL.

I published the bill as finally passed April 5th, and signed by the Governor April 17th, in the April "Red Back." It has not, to this date, May 10th, appeared in any other journal in Texas. This is what newspaper men call a "scoop." I reproduce it this month because of an error of omission by the proof reader, whereby four of the branches on which examinations are to be held were omitted, and some other, but minor, errors; and because, too, I am besieged with requests for copies, and the April issue is exhausted. Because, too, I am daily in receipt of letters asking me to "explain" the bill. It is ambiguous, involved, and, in some places, as clear as mud. It will require an expert opinion—that of the Attorney General—to explain it. For instance: What constitutes a school of medicine,—and how many "schools" are there in Texas—and, consequently, to be represented on the Board; and what will constitute a "majority," as the bill does not say,—"a majority" of the whole, or a "majority" of one "school" over another?

But it is not my intention to criticise it, but to endeavor to explain it, and forestall the flood of inquiries pouring in on me.

It seems to have created consternation, or, at least, a feeling of unrest amongst the older physicians, who, it seems, are to be rounded up,—for record,—and many are asking if they will have to be examined? I will do my darndest to explain; though it looks like having to explain a joke.

*Imprimis.* Notwithstanding the emergency clause, the law does not go into effect until July 17th, ninety days after the Governor signed it, because, vote on final passage, was *viva voce,* and not by yeas and nays.

The Governor has not appointed the Board, and may not do so before July 17th. The Board will meet and elect Secretary and President,—notice of which will be given in the "Red Back," as well as of time and place of first meeting. Of course, it will give the personnel of the Board, and no doubt it will be, like Joseph's coat, of many colors; a thing of beauty and a joy for ever. How delightful for ye "brethren" to dwell together. There will be "harmony" to spare; fish, flesh and fowl and a choice lot of good red herring—what the French call a *pot pourri* (a stink pot) or a "jamboree."

All practicing physicians who, prior to January 1, 1891, registered diploma or certificate, must procure from the clerk of the court, where they registered, an official certificate of such registration. They can do this at any time within a year. This certificate is to be sent to the Secretary of the new Board, when such Board shall have been appointed, accompanied by a fee of 50 cents. A record of all legally qualified physicians; i. e., those who, having complied with the law prior to January 1, 1891, will thus have been obtained by the Board. It is a kind of taking stock of all who are exempt from this new law by reason of their having qualified under previous laws.

Those who have thus qualified will not, of course, have to be examined; nor will those who registered between January 1, 1891, and July, 1901, have to be examined; but they will have to "present to the State Board * * * satisfactory evidence that their diplomas were issued from *bona fide* medical colleges of reputable standing, to be decided by the Board of Medical Examiners, before they are entitled to a certificate from the Board." Sec. 6.

Nothing is required of those who have been licensed by the boards—of the law now in force—the three-board law, repealed by this act, except registration.

Everybody else will have to be examined—by regulars, homeos, physio-meds., eclectics, and osteos, and, perhaps, by Dr. Sam. Who knows?

This wonderful bill has created consternation for sure, and will lead to no end of litigation. The homeos are as much opposed to it as are the ethical of the regular school.

